# Anorexia nervosa artefacta

**DOI:** 10.1186/2050-2974-1-S1-P2

**Published:** 2013-11-14

**Authors:** Yoko Asakawa, Jesuina Noronha, Phillip Bergman

**Affiliations:** 1 Department of Paediatrics, Monash Childrens Hospital, Australia

## 

A 14 year old girl presented with an 8 month history of increasing weight loss, peaking at approximately 10%; and disordered eating, increasing pallor, intermittent fevers and lethargy on the background of a complex social situation, anxiety issues and known congenital heart disease. This occurred on the background of known previous issues regarding desire for thinness in her mother. An outpatient referral had already been made by the GP for concerns of weight loss and food restriction. At presentation she was a pale, sick, emaciated and frail looking adolescent girl. She was afebrile with tachycardia (140 bpm), tachypnoiec (RR 40 breath per min), normotensive with no significant postural drop. Further careful physical examination revealed a cardiac murmur with hyperkinetic apex beat, tender hepatosplenomegaly, red splinter haemorrhages and red macular lesions on her neck, arms and abdomen. Echocardiogram confirmed extensive vegetative lesions consistent with infective endocarditis and she was transferred for cardiac surgery. As her acute issues were being managed, the aetiology of her weight loss was explored and the possible co-existence of an eating disorder was thoroughly assessed but found not to be implicated in this case.

